# A Novel High-Content Phenotypic Screen To Identify Inhibitors of Mitochondrial DNA Maintenance in Trypanosomes

**DOI:** 10.1128/AAC.01980-21

**Published:** 2022-02-15

**Authors:** Migla Miskinyte, John C. Dawson, Ashraff Makda, Dahlia Doughty-Shenton, Neil O. Carragher, Achim Schnaufer

**Affiliations:** a Institute of Immunology and Infection Research, University of Edinburgh, Edinburgh, United Kingdom; b Cancer Research UK Edinburgh Centre, Institute of Genetics and Cancer, University of Edinburgh, Edinburgh, United Kingdom; c MRC Centre for Reproductive Health, The Queen's Medical Research Institute, University of Edinburgh, Edinburgh, United Kingdom

**Keywords:** high-throughput screening, high-content screening, trypanosomatids, kinetoplast, kDNA, mitochondria, *Trypanosoma brucei*, mtDNA

## Abstract

Kinetoplastid parasites cause diverse neglected diseases in humans and livestock, with an urgent need for new treatments. The survival of kinetoplastids depends on their uniquely structured mitochondrial genome (kDNA), the eponymous kinetoplast. Here, we report the development of a high-content screen for pharmacologically induced kDNA loss, based on specific staining of parasites and automated image analysis. As proof of concept, we screened a diverse set of ∼14,000 small molecules and exemplify a validated hit as a novel kDNA-targeting compound.

## INTRODUCTION

Kinetoplastids cause diverse, life-threatening diseases in humans and their livestock, namely, African sleeping sickness (1), Chagas disease (2), and the leishmaniases (3) in humans and animal trypanosomiasis in livestock (4). These diseases particularly affect populations in low- and middle-income countries in many parts of the world. Currently available drugs are unsatisfactory because they cause severe, sometimes lethal side effects, they are difficult to administer, and resistance continues to emerge, necessitating the development of novel antikinetoplastid therapies (5, 6).

Although kinetoplastids have evolved distinct methods of infection and host immune evasion, they all share a unique biological feature: the organization of their mitochondrial DNA (mtDNA; referred to as kDNA in these organisms) in a peculiar structure that gives these organisms their name: the kinetoplast (7). The kDNA is extremely complex, containing hundreds of different classes of “guide RNA”-encoding minicircles of variable copy number, which are essential for posttranscriptional RNA editing in these organisms (8–10). Together with dozens of maxicircles, which are the equivalent of mtDNA in other eukaryotes and encode subunits of the respiratory chain, F_1_F_o_ ATP synthase, and mitoribosomes, thousands of minicircles form an interlinked network structure. The kDNA is thus intrinsically different from mammalian mtDNA, is essential for parasite survival (9, 11), and is a validated target for some current antitrypanosomatid therapies (12–15), making it an attractive target for the discovery of new, improved drugs (16, 17).

Uniquely among kinetoplastids, the sole function of kDNA in bloodstream-form Trypanosoma brucei is the production of subunit α of F_1_F_o_ ATPase (18), which, in this stage of the parasite’s life cycle, operates in reverse to maintain the mitochondrial membrane potential (19). The respiratory chain and oxidative phosphorylation—classical mitochondrial functions—are not functional in bloodstream stage T. brucei. Facilitated by this limited function, kDNA-independent mutants that cause trypanosomiasis in animals have evolved in T. brucei subspecies (18, 20, 21). Typically, kDNA independence in T. brucei is caused by a mutation in the nuclearly encoded subunit γ of the mitochondrial F_1_F_o_ ATPase (18). Importantly, kDNA independence has never been reported for those kinetoplastid parasites of humans and livestock that are currently responsible for the greatest disease and economic burdens by far, i.e., *Leishmania* spp., Trypanosoma cruzi, Trypanosoma vivax, and Trypanosoma congolense. This remains the case despite decades of use of ethidium bromide (EtBr) and isometamidium chloride (phenanthridine compounds that strongly affect kDNA) for the treatment of African animal trypanosomiasis (13–15, 22–24). Loss of kDNA apparently cannot be compensated for in these species, either because additional kDNA-encoded genes are essential (clearly the case for *Leishmania* spp. and T. cruzi, which depend on a functional respiratory chain throughout their life cycles [25]) or because the mutations in F_1_F_o_ ATPase γ that can compensate for the loss of kDNA in bloodstream-stage T. brucei are not functional in these species. Novel antitrypanosomatid therapies based on inhibition of kDNA maintenance are therefore attractive (16, 17).

Drug discovery efforts are typically either phenotypic or target based (26, 27). While target-based campaigns have dominated efforts for decades, they often fail to produce new therapeutic molecules due to the challenge of translating promising results from reductionist biochemical and cellular assays into robust efficacy in more-complex *in vivo* models (28). In contrast, phenotypic screens are often more time-consuming and expensive, and the mode(s) of action behind any identified hits is usually unknown (28). However, the two approaches are complementary and can be used synergistically to fast-track the identification of target-specific compounds that can enter the cell and reach the associated intracellular organelles to induce the desired effect. This paper describes the design, implementation, and validation of a phenotypic high-content screen (HCS) with automated image analysis for the discovery of hit compounds that specifically target kDNA maintenance, using Trypanosoma brucei brucei (referred to below as T. brucei), a causative agent of animal trypanosomiasis, as a model system.

### High-throughput screen (HTS) design and image analysis.

To enable the discovery of target-specific compounds, our phenotypic screen uses a genetically engineered kDNA-independent bloodstream form T. brucei cell line that tolerates kDNA loss due to an L262P mutation in the nuclearly encoded γ subunit of the mitochondrial F_1_F_o_ ATPase (18). Nonspecific cytocidal or cytostatic compounds, or more-general inhibitors of mitochondrial function, which would be more likely to cause side effects in the host, can be readily identified in this genetic background.

**FIG 1:**
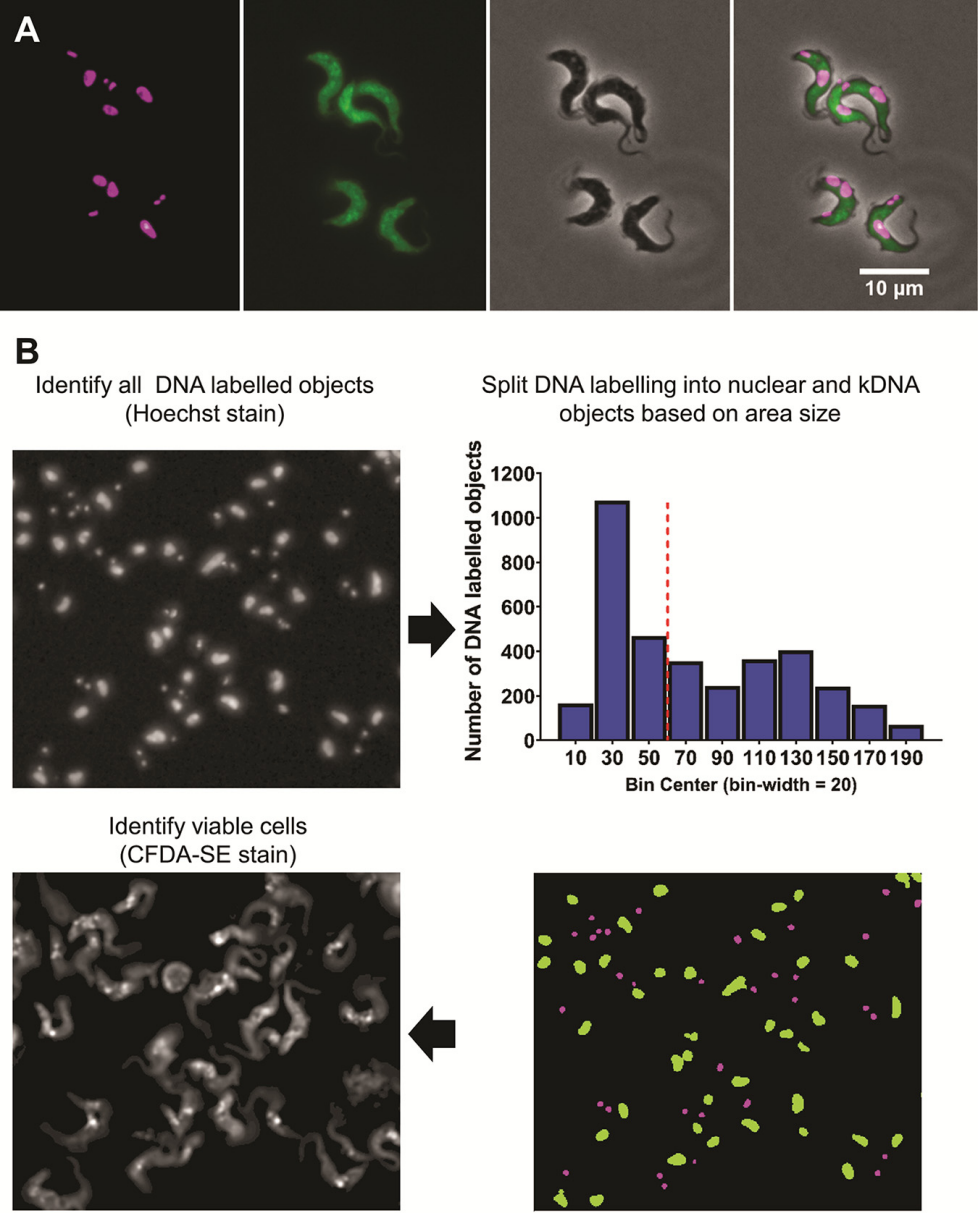
High-content screening strategy to identify compounds inhibiting kDNA maintenance in T. brucei. (A) Representative fluorescence microscopy images of T. brucei using the HCS staining protocol. Shown, from left to right, are Hoechst 33342 staining of trypanosome nuclei and kDNA (in magenta), trypanosomes stained with the cytoplasmic viability stain CFDA-SE (in green), a phase-contrast image, and a merged image. (B) Schematic representation of the image analysis pipeline using CellProfiler. (Top left) First, nuclei and kDNA were identified from the Hoechst 33342 staining. (Top right) Next, nuclei and kDNA were separated by classifying stained objects according to area size. The area size of a nucleus was ≥60 in arbitrary units, and that of kDNA was <60; the bin width was 20, with the bin center ranging from 0 to 200. (Bottom right) Nuclei are shown in green and kDNA in magenta. (Bottom left) Finally, viable cells were identified using the cytoplasmic viability stain CFDA-SE. Each well was imaged at four different, nonoverlapping positions.

Our HCS has been optimized for use in a high-throughput 384-well format (V-bottom; item no. 781280; Greiner Bio-One). We used a Biomek FX liquid handler (Beckman) to dilute all compounds and subsequently added T. brucei L262P cells by using a Viaflo multiwell plate liquid handler (Integra) in a class II biosafety cabinet. Briefly, 2.5 μl compound (at a 200 μM concentration in culture medium with 2% dimethyl sulfoxide [DMSO]) was added to each well. Subsequently, 47.5 μl of parasite culture in complete HMI-9 medium (29), supplemented with 20% (vol/vol) fetal calf serum, was seeded at 50 cells per well, giving a total volume of 50 μl with 1 × 10^3^ cells/ml and a final compound concentration of 10 μM. The plates were incubated under an atmosphere of 5% CO_2_ at 37°C for 4 days (30). Following incubation, the cells were stained first with the cytoplasmic viability stain 5(6)-carboxyfluorescein diacetate succinimidyl ester (CFDA-SE; CAS no. 150347-59-4) at 10 μM for 15 min at 37°C and then, without any washing steps, with Hoechst 33342 nucleic acid stain at 1 μg/mL for 5 min at 37°C. Subsequently, cells were fixed with 2% (wt/vol) (final concentration) formaldehyde, with vigorous mixing to avoid clumped cells, a step that is crucial for subsequent image analysis (Fig. 1A). After 24 h of fixation at 4°C, cells were washed three times with phosphate-buffered saline by centrifuging plates at 1,000 × *g* for 1 min to remove any remaining dye. Loss of cells during washing steps was minimized by using V-bottom plates and carefully adjusting fixed pipette positions for the Biomek FX liquid handler. The cells were then transferred to 384-well F-bottom plates for imaging (item no. 781986; Greiner Bio-One). The plates were centrifuged at 1,000 × *g* for 5 min prior to image acquisition at ×40 magnification using an automated ImageXpress Micro XLS (Molecular Devices) HCS system. Each well was imaged across four different fields of view using a 4′,6-diamidino-2-phenylindole (DAPI) filter set for the Hoechst 33342 stain and a fluorescein isothiocyanate (FITC) filter set for CFDA-SE. Image analysis was performed using CellProfiler software, version 3.1.9 (31). Briefly, nuclear DNA and kDNA were identified based on the area sizes of Hoechst 33342-positive objects, and viable cells were identified using the FITC channel (Fig. 1B, see also Fig. S1 in the supplemental material).

### HCS performance validation and pilot screen.

Plates (*n* = 2) were prepared as described above, with even-numbered columns containing a negative control (0.1% DMSO) and odd-numbered columns containing 10 nM EtBr (in 0.1% [vol/vol] DMSO), a known inhibitor of kDNA maintenance, as a positive control (14). A “robust” *Z′* assay performance score of 0.725 was calculated (32, 33), indicating excellent performance (34).

**FIG 2:**
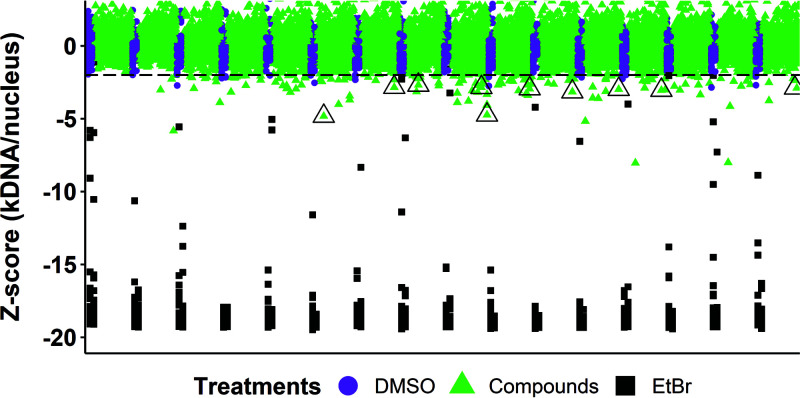
HCS results and hit selection. Tested compounds were ranked based on the decrease of the kDNA/nucleus ratio in imaged wells (*Z*-score, <–2 [delineated by the dashed black line]), resulting in 152 hits (see also Table S1). Images of the top 50 hits (based on ranking by the decrease in the kDNA/nucleus ratio) were then reexamined using ImageJ software. Ten compounds (symbols enclosed in triangles) were selected for follow-up analysis, based on the observation of a complete loss of kDNA and on commercial availability.

To test the ability of our HCS to identify novel inhibitors of kDNA maintenance, 13,486 compounds were screened, from a diverse set of chemical libraries: Prestwick Chemical Library (1,280 compounds; Prestwick Chemical), Screen-Well PKE library (consisting of 53 protease, 80 kinase, and 43 epigenetic inhibitors; Enzo Biochem), and BioAscent 12K diverse chemical libraries (11,970 compounds; BioAscent Discovery Ltd.). The Prestwick Chemical Library was designed to represent the broad pharmacological diversity of all FDA-approved small-molecule drug classes and consists of drugs with known pharmacological, toxicological, and pharmacokinetic properties to support the repurposing of existing drugs. The BioAscent 12K compound library is a subset representing the chemical diversity of a 125,000-compound parent library, enabling subsequent expansion of screening hits to explore structure-activity relationships. All compounds were screened at a final concentration of 10 μM in 0.1% (vol/vol) DMSO in a 384-well format, where the first four columns had alternating positive (EtBr) and negative (DMSO) controls. Additionally, the PKE and Prestwick Chemical libraries were also screened at a lower final concentration of 1 μM, because both libraries have been reported to lead to the identification of potent inhibitors in different phenotypic screening assays at this lower dose, which may better reflect on-target activity rather than the off-target activity observed at higher doses (35, 36). The screens were performed in five batches (48 plates in total), with a “robust” *Z′* assay performance score (33) ranging from 0.63 to 0.9 between batches. The HCS identified 152 compounds with a reduced ratio of kDNA per nucleus (*Z*-score, <–2) (Fig. 2; Table S1). Separate results for nucleus and kDNA counts for all wells are shown in Fig. S2.

### Hit validation.

**FIG 3:**
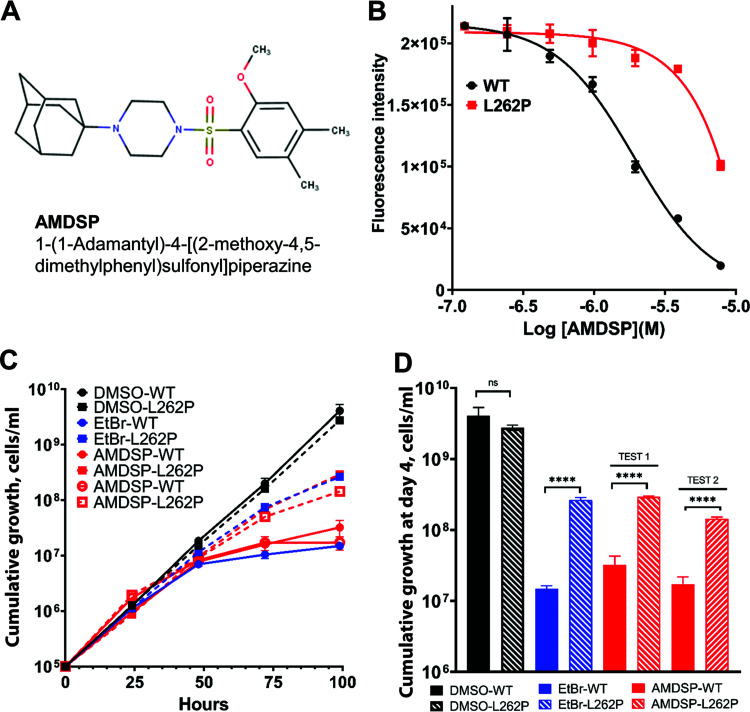
Hit validation. (A) Structure of AMDSP (BCC0052412). (B) Dose-response curves for the effects of AMDSP on the growth of kDNA-dependent (WT) (black squares) and kDNA-independent (L262P) (red squares) bloodstream-form T. brucei. (C) Cumulative growth curves of bloodstream-form T. brucei cells cultured in the presence (dashed lines) and absence (solid lines, filled circles) of 12.5 μM AMDSP (red) or 10 nM EtBr (blue). Growth curves in the presence of solvent only (0.1% DMSO) are shown as controls (black). Cell numbers were determined with a Coulter counter. (D) Comparison of cumulative cell numbers in WT and L262P cells after 96 h as shown in panel C. Asterisks indicate significant differences (****, *P < *0.00005) by the Student unpaired *t* test. All experiments were performed in triplicate; in addition, the effects of AMDSP on WT and L262P cells were tested on two separate occasions (test 1 and test 2).

**FIG 4:**
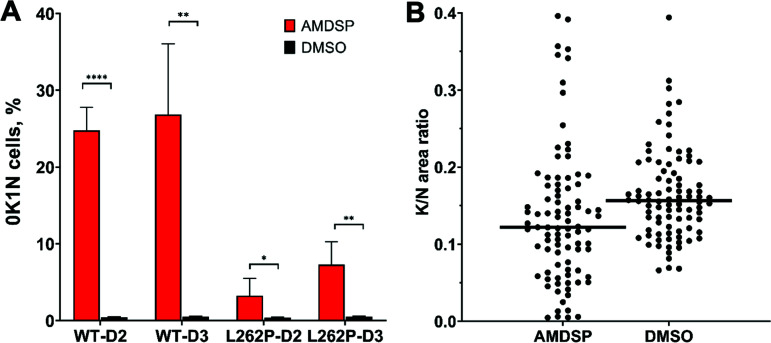
AMDSP affects kDNA maintenance. (A) Loss of kDNA (0K1N cells are cells with no kinetoplast and one nucleus) assessed by DAPI staining and microscopy after 2 days (D2) and 3 days (D3) of culturing in the presence or absence of 12.5 μM AMDSP. The statistical significance of differences was assessed with the Student unpaired *t* test (*, *P* ≤ 0.05; **, *P* ≤ 0.01; ***, *P* ≤ 0.001). (B) The relative amount of kDNA in 1K1N cells (cells with one kinetoplast and one nucleus) after 2 days of culturing was assessed by DAPI staining and quantitation of kinetoplast versus nucleus fluorescence intensity. The statistical significance of differences (*P* < 0.001) was assessed with the Mann-Whitney test for AMDSP at 12.5 μM in 0.1% DMSO (*n* = 90) versus 0.1% DMSO alone (*n* = 90). All experiments were performed in triplicate.

For the top 50 compounds, based on ranking by the kDNA/nucleus ratio (excluding all compounds that had fewer than 50 DNA objects per well) and a *Z*-score of <–2 (Table S1), we manually reviewed the microscopy images for evidence of complete kDNA loss. Ten candidates (Table S1) were cherry-picked for follow-up analysis based on consistently observed loss of kDNA from cells treated with these compounds and on their commercial availability. Purchased compounds were dissolved in DMSO, and their potency against wild-type (WT) T. brucei cells was evaluated using an adapted 3-day alamarBlue method (18). Only two compounds, (*S*)-propranolol hydrochloride and 1-(1-adamantyl)-4-[(2-methoxy-4,5-dimethylphenyl)sulfonyl]piperazine (AMDSP; BioAscent code BCC0052412) were sufficiently potent at the highest concentration that could be tested (due to limited solubility in water) to permit the calculation of 50% inhibitory concentration (IC_50_) values of 16 to 22 μM and 1.6 to 2.3 μM, respectively, for WT cells (95% confidence intervals) (Table S1, second tab); the other eight compounds did not significantly affect the growth of WT cells in the alamarBlue assay. Next, we assessed the specificities of these two compounds as inhibitors of kDNA maintenance. Such specificity is indicated by the selectivity for killing of kDNA-dependent (“WT”) and kDNA-independent (“L262P”), but otherwise isogenic, T. brucei cells. The most specific compound reported to date is EtBr, with a selectivity index of ∼300 in the modified alamarBlue assay (14). One of the two compounds tested, AMDSP (Fig. 3A), reproducibly affected the viability of WT T. brucei cells at a concentration lower than that for L262P cells (Fig. 3B). The IC_50_ for WT cells was 1.9 μM, while that for L262P cells was estimated to be in the range of 8 μM (the value could not be determined more precisely due to poor compound solubility in DMSO at stock concentrations higher than 12.5 mM). To investigate the time required for AMDSP to affect growth, we performed growth curves for WT and L262P cells at a final compound concentration of 12.5 μM in 0.1% (vol/vol) DMSO (Fig. 3C and D). After 3 days of AMDSP treatment, the growth of WT cells was much more severely inhibited than that of L262P cells. No growth was observed between days 3 and 4 for one of the WT replicates (Fig. 3C, open red circles). The cumulative growth curve for the other replicate indicated a slight increase in cell numbers between days 3 and 4 (Fig. 3C, filled red circles). However, by microscopy, we found no intact and motile cells of either WT replicate after 4 days, even after concentration of the culture by centrifugation, while L262P cells survived. Hence, it is more likely that the apparent increase for one of the WT replicates was caused by counting of cell debris in the Coulter machine. Moreover, we observed a substantial increase in the proportion of cells with complete loss of kDNA (0K1N cells) in WT and L262P cells after 2 or 3 days of exposure to 12.5 μM AMDSP (Fig. 4A). Interestingly, loss of kDNA was more severe for WT cells than for L262P cells. This could suggest reduced uptake of AMDSP in L262P cells, perhaps caused by the lower mitochondrial membrane potential in these cells (37). In further support of an effect of AMDSP on kDNA maintenance, for the proportion of WT cells that had retained at least some kDNA after AMDSP treatment, we observed a significant reduction in kDNA size compared to control cells (Fig. 4B), while the size of the nucleus was not affected (Fig. S3).

Taken together, these data confirm that an important part of the mode of action of AMDSP in trypanosomes is interference with kDNA maintenance. The data are consistent with the dynamics of growth inhibition and the effects on kDNA of other compounds that preferentially target this structure, such as EtBr (13, 37, 38), although, unsurprisingly, the potency and selectivity of this primary hit are much lower. Nonetheless, AMDSP may represent a promising starting point for hit-to-lead development. The compound is composed of piperazine, benzene, and adamantane rings with a tertiary sulfonamide group. Adamantane derivatives, such as the well-studied drug amantadine (1-amino-adamantane), show good pharmacokinetics in humans, are licensed drugs for the treatment of Parkinson’s disease, and in the past had been used for the treatment of influenza until the emergence of resistance halted their application for this purpose (39). Moreover, the discovery of aminoadamantane derivatives with trypanocidal activity (40) has spurred recent efforts to develop more-potent adamantane-benzene derivatives (41). Piperazine-based anthelminthic drugs (42) have also attracted interest in drug design studies because of their trypanocidal activity (43). The exact mechanism(s) by which the derivatives described affect trypanosomatids remains unknown, but on the basis of our findings, effects on kDNA should be explored. Furthermore, similarity searches of the full BioAscent library with AMDSP suggest as many as 150 related compounds that could be tested against trypanosomatids in the future to explore structure-activity relationships.

### Identification of other antitrypanosomatid compounds with unknown modes of action.

In addition to a novel inhibitor of kDNA maintenance, we identified compounds that strongly affected the viability of the kDNA-independent T. brucei cell line used for screening and that therefore must act via a different mechanism. To find such trypanocidal or trypanostatic hits, we first corrected for positional growth effects in our plates using the median polish normalization method (44, 45) (Fig. S4). Median polish normalization was performed with Spotfire software (PerkinElmer) by using the High Content Profiler package to remove row and column biases. This method uses the row and column medians to identify the row and column effects on the data. We then scored for hits affecting T. brucei viability based on fewer than 10 total nuclei per image with *Z*-scores of <–1. We identified 337 hits, corresponding to a hit rate of 2.5% (Table S2; Fig. S5, left). These include 31 compounds from the Prestwick Chemical Library that inhibited trypanosome growth at both 10 μM and 1 μM (Table S2, double underlining), suggesting a good starting potency for any lead development efforts. Incidentally, among the compounds tested in our proof-of-concept screen were nine with known antitrypanosomatid activity (46). Seven of these compounds were among the hits with a *Z*-score of <–1 (highlighted in Table S2; Fig. S5, right). This further confirms the robustness of our HCS assay and suggests that, as an additional benefit, the outputs from this assay could also be used for the identification of antitrypanosomatid compounds with a mode of action unrelated to kDNA maintenance.

In conclusion, we successfully established and validated a scalable, kDNA maintenance-based phenotypic HCS with automated image analysis, using an engineered kDNA-independent T. brucei cell line as a kinetoplastid model system. A proof-of-concept screen of diverse small-compound libraries identified and validated a novel compound affecting kDNA maintenance in T. brucei. To the best of our knowledge, this is the first HCS specifically designed to identify inhibitors of kDNA maintenance. Furthermore, we identified other antitrypanosomatid compounds with activity in the low micromolar range (but with unknown molecular targets) that could be useful starting points for trypanosomatid drug development. In the future, the screen could be further optimized by trying to address the positional growth effects in plates and by developing machine learning algorithms that can lower the rate of false-positive hits and detect subtler changes in kDNA, nuclear DNA, and cell morphology.
