# Supplementary material for: A Novel High-Content Phenotypic Screen To Identify Inhibitors of Mitochondrial DNA Maintenance in Trypanosomes
Source: Antimicrob Agents Chemother. 2022 Feb 15;66(2):e01980-21. doi: 10.1128/AAC.01980-21 (PMC8846439; doi:10.1128/AAC.01980-21)
Supplement: Supplemental file 1 — Supplemental figures and references. Download AAC.01980-21-s0003.pdf, PDF file, 6.1 MB [file aac.01980-21-s0003.pdf]

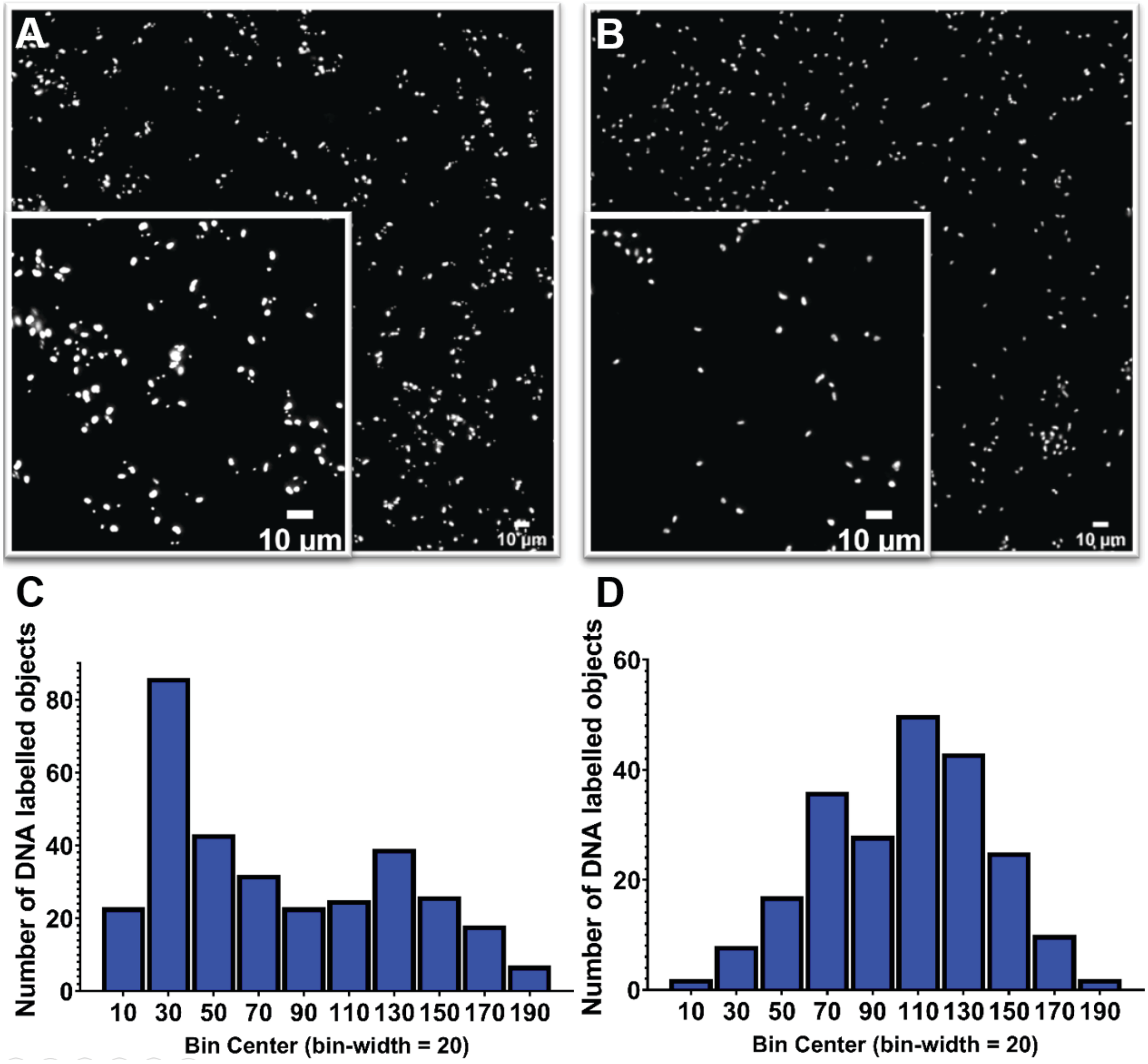

Figure S1. Fluorescent images of *T. brucei* DNA in DMSO and EtBr treated samples. Hoechst 33342 staining of trypanosome nuclei and kDNA in cells treated with **(A)** DMSO and **(B)** EtBr. Distribution of DNA content as measured by quantification of area size using CellProfiler in **(C)** DMSO and **(D)** EtBr treated samples (area size in arbitrary units; bin width = 20, with bin centre ranging from 0 to 200).

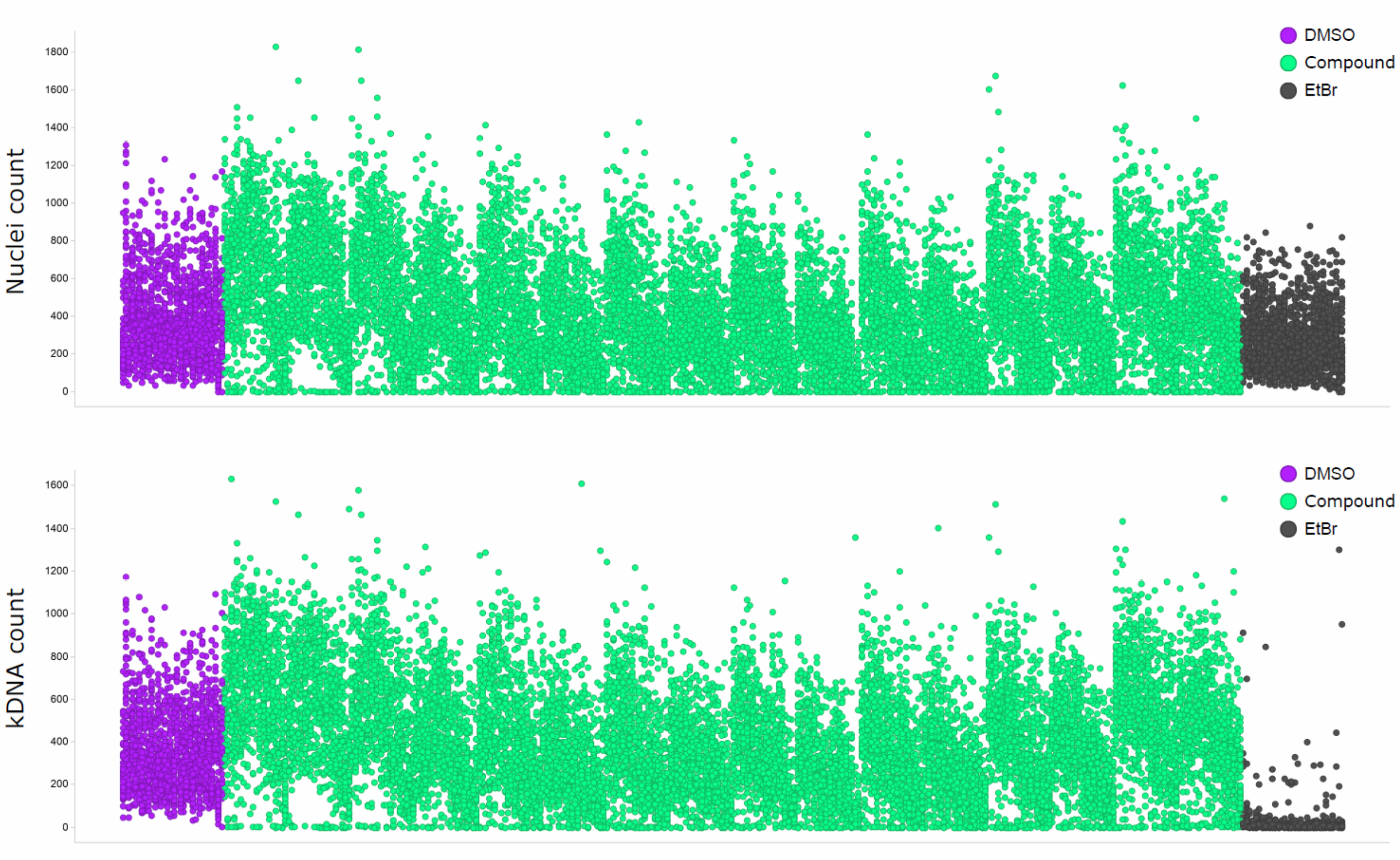

Figure S2. Summary of results from the HCS screen. Nuclei (top) and kDNA (bottom) counts per well. Each dot represents the median aggregated values from four separate images for this well. EtBr, ethidium bromide.

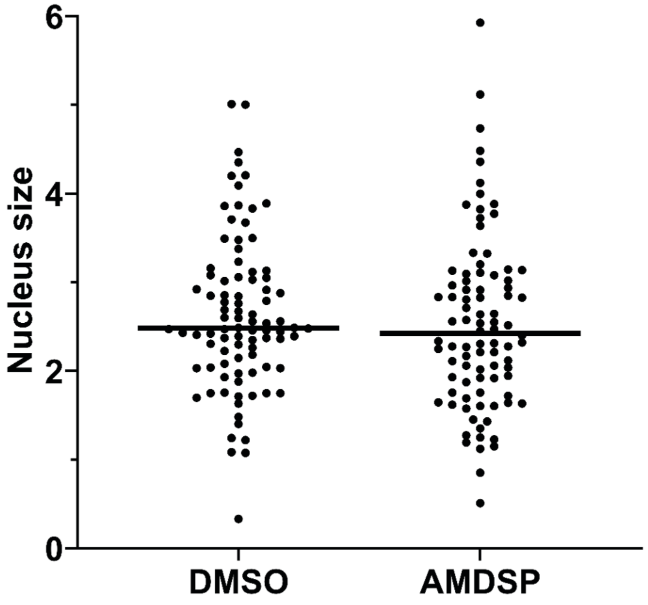

Figure S3. Nuclear size in DMSO and AMDSP treated cells. Size of nuclei in trypanosomes after 2 days of culturing WT cells in 0.1% DMSO (n = 90) or 12.5 μM AMDSP (n = 90) was assessed by DAPI staining and quantitation of fluorescence intensity using ImageJ software. There was no significant difference between treatment groups, Mann-Whitney test; $P > 0.05$. All experiments were performed in triplicate.

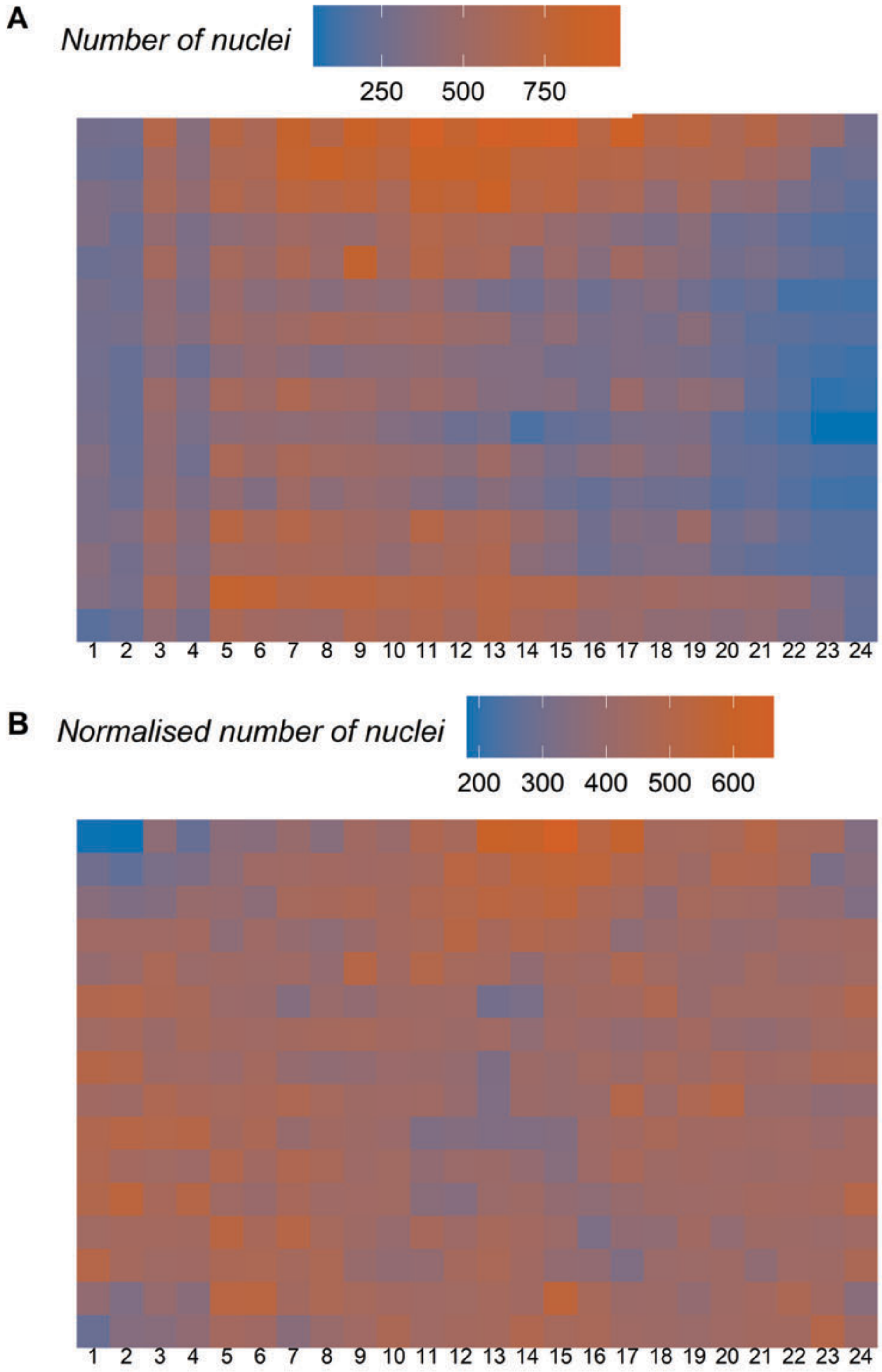

Figure S4. Normalisation of plate edge effects. Nuclei numbers per well are indicated as heat maps. To correct for an apparent edge effect (presumably caused by temperature and/or gas gradients during incubation), raw values for the number of counted nuclei per well (A) were normalised using a median polish method (B). DMSO controls were plated in the first and third columns, while EtBr controls were in the second and fourth columns of each plate.

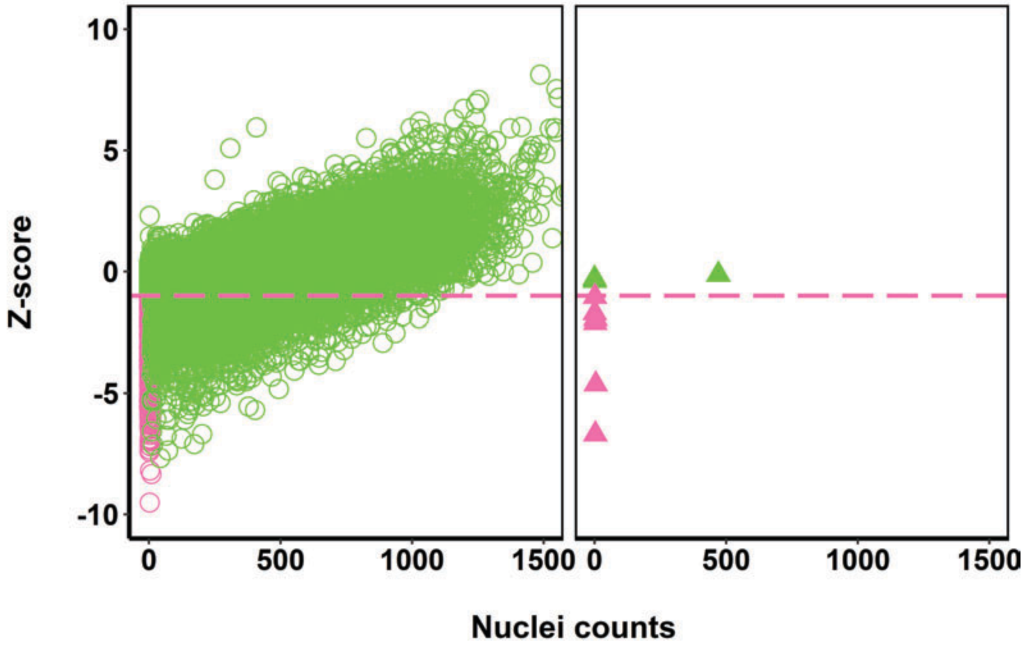

Figure S5. Compounds affecting viability of kDNA-independent *T. brucei*. The graph on the left highlights 33 compounds (pink circles; also listed in Table S2) that affected viability of kDNA-independent *T. brucei*, based on reduction in total nuclei counts (0 - 10 nuclei per well) and a Z-score < -1 (cut-off shown in dashed pink line), and after normalisation for plate edge effects (Figure S4). Nine known anti-trypanosomatid compounds in the test set are plotted as triangles in the graph on the right. Two anti-trypanosomatid compounds did not meet the cut-off criteria (green triangles).

Supplemental References

1. Dean S, Gould MK, Dewar CE, Schnaufer AC. 2013. Single point mutations in ATP synthase compensate for mitochondrial genome loss in trypanosomes. Proc Natl Acad Sci U S A 110:14741–14746.
